# MicroRNA-1305 Inhibits the Stemness of LCSCs and Tumorigenesis by Repressing the UBE2T-Dependent Akt-Signaling Pathway

**DOI:** 10.1016/j.omtn.2019.04.013

**Published:** 2019-04-22

**Authors:** Xiaoyong Wei, Xiaolong You, Jianlong Zhang, Cuncai Zhou

**Affiliations:** 1 Department of Hepatobiliary Surgery, Jiangxi Cancer Hospital, Nanchang 330029, Jiangxi Province, P.R. China

**Keywords:** microRNA-1305, ubiquitin-conjugating enzyme E2T, Akt-signaling pathway, liver cancer stem cells, hepatocellular carcinoma, self-renewal, stemness, tumorigenicity

## Abstract

MicroRNAs (miRNAs) are involved in the maintenance of the cancer stem cell (CSC) phenotype by binding to genes and proteins that modulate cell proliferation and/or cell apoptosis. In our study, we aimed to investigate the role of miR-1305 in the proliferation and self-renewal of liver CSCs (LCSCs) via the ubiquitin-conjugating enzyme E2T (UBE2T)-mediated Akt-signaling pathway. Differentially expressed genes in human hepatocellular carcinoma (HCC) were obtained by *in silico* analysis. The relationship between miR-1305 and UBE2T was verified by dual luciferase reporter gene assay. qRT-PCR and western blot analysis were performed to determine the expression of UBE2T, the Akt-signaling pathway, and stemness-related factors in LCSCs. In addition, miR-1305 disrupted the activation of the Akt-signaling pathway by targeting UBE2T, and, ultimately, it repressed the sphere formation, colony formation, and proliferation, as well as tumorigenicity of LCSCs. In summary, miR-1305 targeted UBE2T to inhibit the Akt-signaling pathway, thereby suppressing the self-renewal and tumorigenicity of LCSCs. Those findings may provide an enhanced understanding of miR-1305 as a therapeutic target to limit the progression of LCSCs.

## Introduction

As the most common primary malignancy in the liver, hepatocellular carcinoma (HCC) is highly prevalent around the globe. HCC ranks as the fifth most common cancer in males and ninth in women. It is also the second leading cause of cancer-related mortality in China.^1^, ^2^ In addition, eastern and southeastern Asia regions are known to exhibit a high incidence of HCC in the male population.^3^ Contrary to other malignancies, HCC patients have an array of treatment options, including surgical resection, chemotherapy, and transplantation.^4^ Although systemic treatment is employed clinically, the mortality rate of HCC still remains to be high because of recurrence and drug resistance.^5^ In general, cancer stem cells (CSCs) are dormant or slowly cycling tumor cells, which are capable of reconstituting tumors.^6^ CSCs have been observed in solid tumors with a range of stem cell markers, including CD133. In addition, CD133^+^ HCC cells isolated from HCC cell lines and xenograft tumors possess boosted colony formation, proliferation, and tumorigenicity *in vivo*.^7^

Ubiquitin-conjugating enzyme E2T (UBE2T, also called HSPC150), a member of the E2 family, was initially found in a case of Fanconi anemia.^8^ UBE2T was demonstrated to be amplified in human fibroblasts after serum stimulation.^9^ Importantly, the UBE2T gene, located at 1q32.1, was reported to be overexpressed in several cancers, such as prostate cancer and gastric cancer.^10^, ^11^ MicroRNAs (miRNAs), a group of small non-coding RNAs, have been proposed as useful biomarkers for disease diagnosis and prognosis, especially for cancer-related diseases.^12^ Mature miRNAs are known to modulate genes through complementary interactions with the 3′ UTR of mRNA, which lead to the degradation of mRNA or suppression of the protein translation.^13^ Specifically, the elevation of miR-1305 reversed the suppressor effect of nicotine on periodontal ligament-derived stem cell proliferation and migration.^14^ Furthermore, the role of miR-1305 in the modulation of pluripotency early differentiation balance and survival of pluripotent stem cells has already been investigated.^15^

In addition, previous evidence strongly indicates the functional correlation between the tumorigenicity of the CD133^+^ CSC with activation of the Akt-signaling pathway.^16^ Strikingly, UBE2T has been verified to be an independent prognostic factor for nasopharyngeal carcinoma. Furthermore, UBE2T enhanced cell proliferation, invasion, and metastasis by activating the Akt/glycogen synthase kinase-3β (GSK3β)-signaling pathway.^17^ Therefore, our study aimed to elucidate the dominant role of miR-1305 and UBE2T in HCC carcinogenesis. We showed that UBE2T was found overexpressed in HCC cell lines and liver CSCs (LCSCs), and we further revealed that UBE2T promoted proliferation and sphere and colony formation of LCSCs through the Akt-signaling pathway. These data indicated that UBE2T is a novel oncogene and a potential therapeutic target for HCC.

## Results

### UBE2T Was Upregulated in HCC Samples

**Figure 1:**
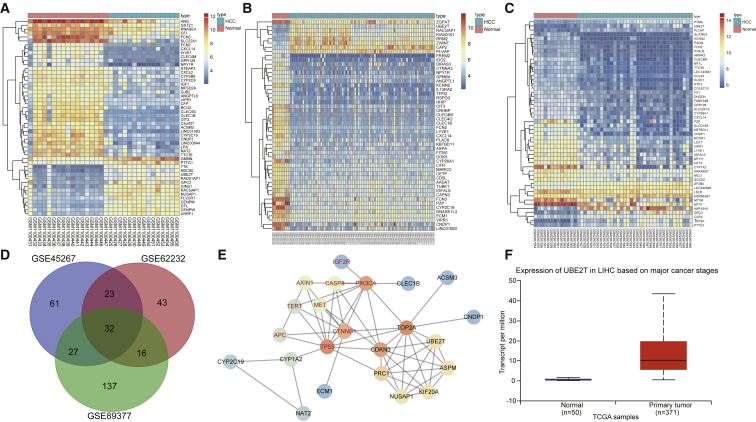
UBE2T Affected the Progression of HCC (A–C) Heatmaps of DEGs in GEO: GSE45267 (A), GSE62232 (B), and GSE89377 (C) microarrays (the abscissa indicates the sample number, the ordinate indicates the gene name, and the left dendrogram indicates gene expression cluster; each small square indicates the expression of one gene in one sample). (D) Venn analysis of DEGs in three HCC microarrays (the three circles represent the top 50 DEGs among the three microarrays, and the middle part indicates the intersection of the three microarrays). (E) The connection analysis between DEGs obtained from three microarrays and 10 known genes (each circle represents the expression of a gene in a sample, the red font represents a known gene in HCC retrieved from the database, the black font represents the DEGs obtained from microarray-based gene expression analysis, and the line between the two genes indicates there was an interaction between the two genes). (F) The expression of UBE2T in the HCC databases of TCGA was analyzed (the horizontal coordinate indicates the sample type, the vertical coordinate represents the expression of UBE2T, the left boxplot represents the expression of UBE2T in the normal sample, and the right boxplot represents the expression of UBE2T in the HCC samples).

Initially, the GEO database was employed in order to retrieve HCC-related microarray data. Then, GEO: GSE45267, GSE62232, and GSE89377 microarrays were obtained. The difference between the HCC samples and the normal samples was analyzed. Finally, a total of 286, 230, and 425 differentially expressed genes (DEGs) were obtained in the GEO: GSE45267, GSE62232, and GSE89377 microarrays, respectively. Figures 1A–1C displayed 50 distinct DEGs in each of the three microarrays. To further screen out DEGs associated with HCC, a Venn analysis of the top 50 of the DEGs in the three microarrays (Figure 1D) was used, which showed that there were 32 genes in the intersection. In addition, 10 known genes related to HCC were obtained from the MalaCards database. The connection analysis between DEGs obtained from the three microarrays and 10 known genes was conducted, and the gene interaction network was constructed (Figure 1E).

The results revealed that, of the 32 DEGs, genes such as UBE2T, TOP2A, and CDKN3 were at the core of the whole gene interaction network. Among these core genes, we noted that UBE2T played an important role in numerous tumor diseases.^18^, ^19^ The expression of UBE2T was further analyzed in The Cancer Genome Atlas (TCGA) database, and the results revealed that the expression of UBE2T significantly increased in HCC samples (Figure 1F), which was consistent with the expression of UBE2T in the HCC expression microarray. So, we intended to further study the role of UBE2T in LCSCs.

### UBE2T Was Highly Expressed in HCC Cell Lines and LCSCs

**Figure 2:**
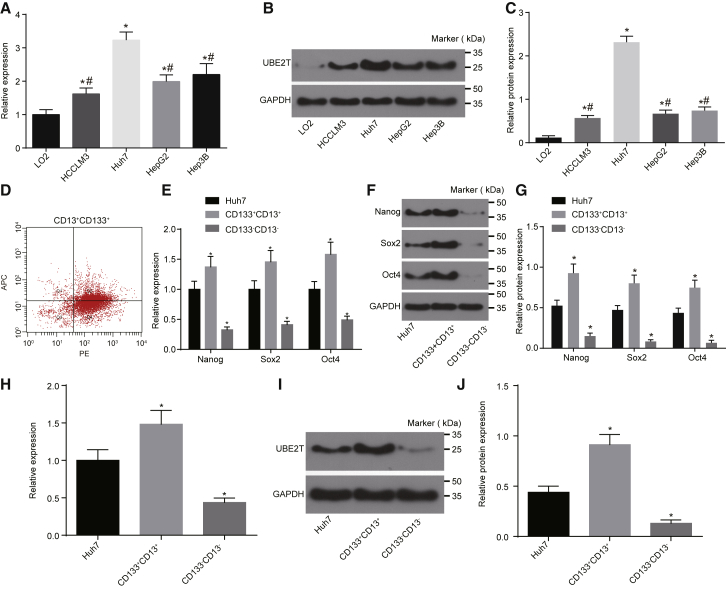
UBE2T Was Highly Expressed in LCSCs (A) The mRNA expression of UBE2T in the HCC cell line and normal hepatic epithelial cell line determined by qRT-PCR. (B) The gray value of the UBE2T protein band in the HCC cell line and normal hepatic epithelial cell line. (C) The protein expression of UBE2T in the HCC cell line and normal hepatic epithelial cell line, determined by western blot analysis. (D) LCSCs sorted in the Huh7 cells by flow cytometry. (E) The mRNA expression of Nanog, Sox2, and Oct4 in Huh7 cells, CD13^+^CD133^+^ cells, and CD13^−^CD133^−^ cells, measured by qRT-PCR. (F) The gray value of Nanog, Sox2, and Oct4 protein bands in Huh7 cells, CD13^+^CD133^+^ cells, and CD13^−^CD133^−^ cells measured by western blot analysis. (G) The protein expression of Nanog, Sox2, and Oct4 in Huh7 cells, CD13^+^CD133^+^ cells, and CD13^−^CD133^−^ cells, measured by western blot analysis. (H) The mRNA expression of UBE2T in Huh7 cells, CD13^+^CD133^+^ cells, and CD13^−^CD133^−^ cells, determined by qRT-PCR. (I) The gray value of UBE2T protein band in Huh7 cells, CD13^+^CD133^+^ cells and CD13^−^CD133^−^ cells determined by western blot analysis. (J) The protein expression of UBE2T in Huh7 cells, CD13^+^CD133^+^ cells, and CD13^−^CD133^−^ cells, determined by western blot analysis. *p < 0.05 versus the LO2 cells; #p < 0.05 versus the Huh7 cells. Statistical data were described as mean ± SD and was compared using one-way ANOVA. The experiment was repeated 3 times, independently.

The expression of UBE2T in the normal hepatic epithelial cell line LO2, along with HCC cell lines HCCLM3, Hep3B, HepG2, and Huh7, was determined by qRT-PCR and western blot analysis. The results showed that the expression of UBE2T was higher in the HCC cell lines compared to the normal hepatic cell line LO2 (all p < 0.05). In addition, UBE2T was found to be expressed differently in 4 kinds of HCC cells, and the order of UBE2T expression from high to low was Huh7, Hep3B, HepG2, and HCCLM3 (Figures 2A–2C).

Furthermore, CD133^+^CD13^+^ and CD133^−^CD13^−^ cells were isolated from Huh7 cells by means of flow cytometry, the results of which revealed that CD133^+^CD13^+^ cells accounted for 44.21% ± 4.42%. The percentage of CD133^−^CD13^−^ cells was 19.3% ± 1.61% (Figure 2D). Moreover, the expressions of Nanog, Sox2, and Oct4 in Huh7 cells were determined by qRT-PCR and western blot analysis. The results showed that the expressions of Nanog, Sox2, and Oct4 were significantly higher in CD133^+^CD13^+^ cells compared to Huh7 cells (p < 0.05). The expression levels of Nanog, Sox2, and Oct4 were noted to significantly decrease in CD133^−^CD13^−^ cells when compared with Huh7 cells (p < 0.05) (Figures 2E–2G). Furthermore, the results showed that the expression of UBE2T was significantly higher in CD133^+^CD13^+^ cells than in Huh7 cells (p < 0.05), while the expression of UBE2T in CD133^−^CD13^−^ cells significantly decreased (Figures 2H–2J) (p < 0.05). Therefore, CD133^+^CD13^+^ cells were selected as subsets of LCSCs for subsequent experimentation.

### Silencing of UBE2T Inhibited LCSC Self-Renewal and Tumorigenicity

**Figure 3:**
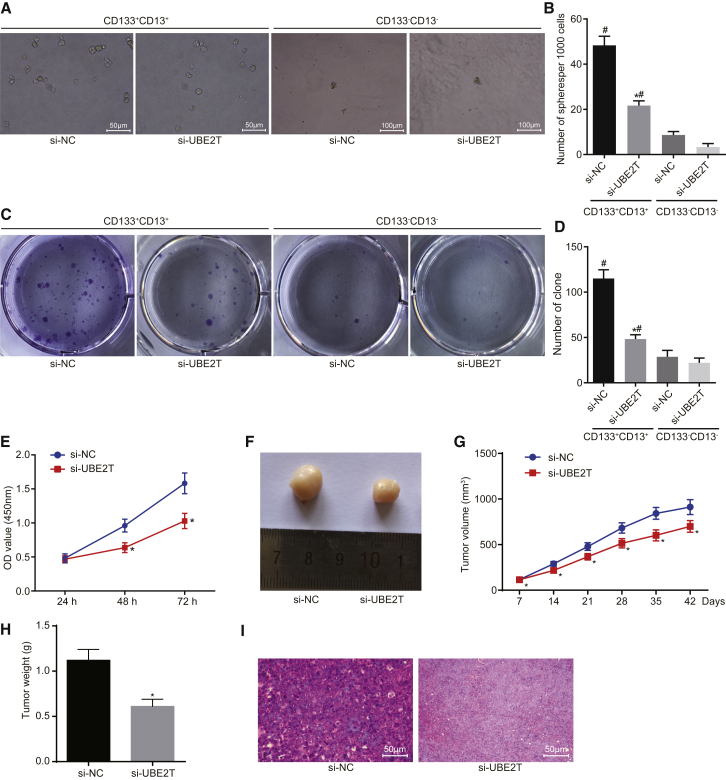
UBE2T Promoted the Tumorigenic Potential of LCSCs (A) Sphere formation ability of LCSCs in the presence of UBE2T silencing (200×). (B) The number of newly formed spheres. (C) Colony formation ability of LCSCs in the presence of UBE2T silencing, determined by soft agar colony formation assay. (D) The number of newly formed colonies. (E) The proliferative ability of LCSCs transfected with UBE2T silencing, assessed by CCK-8 assay. (F) Observation of tumors in nude mice. (G) The tumor volume in nude mice injected with UBE2T silencing-transfected LCSCs, measured by Vernier caliper. (H) The tumor weight in nude mice injected with UBE2T silencing-transfected LCSCs. (I) The pathological characteristics of tumor tissues in nude mice observed by H&E staining (400×). si-NC group, LCSCs transfected with si-NC or nude mice inoculated with si-NC-transfected LCSCs; si-UBE2T mimic group, LCSCs transfected with UBE2T silencing or nude mice treated with UBE2T silencing-transfected LCSCs. *p < 0.05 versus LCSCs transfected with si-NC or nude mice inoculated with si-NC-transfected LCSCs; #p < 0.05 versus CD13^−^CD133^−^ cells. Statistical data were described as mean ± SD. Data between two groups were analyzed by unpaired t test, and data in (E) and (G) were compared by repeated-measures ANOVA. The experiment was repeated 3 times independently (n = 6).

To define the functional significance of UBE2T in LCSCs, we performed *in vitro* and *in vivo* gain-of-function experiments. The LCSCs were transfected with silencer (si)-UBE2T or si-negative control (NC), and the nude mice were inoculated with the transfected cells in order to examine the effects on self-renewal and tumorigenicity of LCSCs. The results of sphere formation assays and soft agar colony formation assay revealed the formation of some new spheres in LCSCs. Silencing of UBE2T caused a significant decrease in the number of newly formed spheres, with irregular shape and weak refraction, as well as freshly formed colonies; CD133^−^CD13^−^ cells presented with a smaller number of formed spheres and formed colonies compared to CD133^+^CD13^+^ cells (Figures 3A–3D). Similarly, the cell counting kit-8 (CCK-8) assay result showed that UBE2T silencing markedly reduced the cell proliferative ability; CD133^−^CD13^−^ cells showed weaker cell proliferative ability compared to CD133^+^CD13^+^ cells (Figure 3E).

The results of animal experiments showed that tumor growth was observed in nude mice since the seventh day. On the 14^th^ day, the tumor volume and weight of mice were noted to significantly decrease by the silencing of UBE2T (Figures 3F–3H). The results of H&E staining showed that, introduced with si-NC, the tumor tissue was in a lump and the nucleus was large and deep stained; while the tumor cells grew vigorously with more mitotic appearance and focal necrosis, the tumor tissue infiltrated the surrounding tissue and could invade muscle tissues in mice. The tumor in nude mice inoculated with UBE2T silencing-transfected cells showed lighter nuclear staining and reduced nuclear division of tumor cells, with extensive necrosis in the tumor tissues and proliferated fibrous tissues around the necrotic area to different degrees (Figure 3I). Collectively, these data indicated that UBE2T silencing could inhibit the self-renewal and tumorigenicity of LCSCs *in vivo*.

### UBE2T Was a Functional Target of miR-1305 in HCC

**Figure 4:**
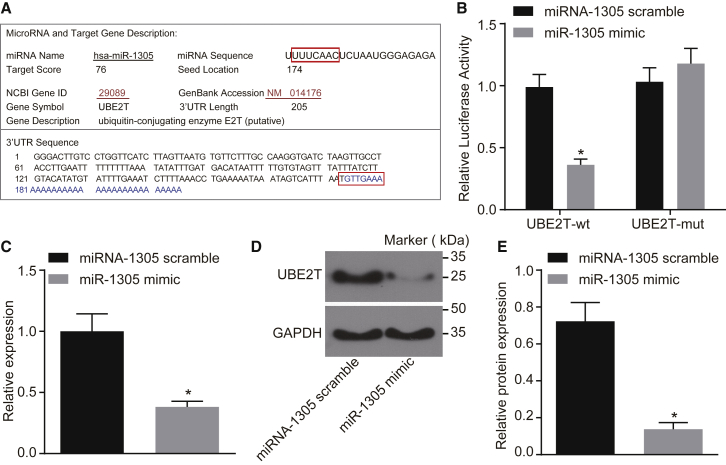
UBE2T Was a Target Gene of miR-1305 (A) The predictive binding site between miR-1305 and UBE2T 3′ UTR. (B) Dual luciferase reporter gene assay for confirmation of the targeting relationship between miR-1305 and UBE2T. (C) The mRNA expression of UBE2T in the presence of miR-1305 mimic, determined by qRT-PCR. (D) The gray value of the UBE2T protein band in the presence of miR-1305 mimic determined by western blot analysis. (E) The protein expression of UBE2T in the presence of miR-1305 mimic, determined by western blot analysis. miR-1305 scramble group, LCSCs transfected with scramble miR-1305; miR-1305 mimic group, LCSCs transfected with miR-1305 mimic. *p < 0.05 versus LCSCs transfected with scramble miR-1305. Statistical data were described as mean ± SD. Data between two groups were analyzed by unpaired t test. The experiment was repeated 3 times independently.

To understand the upstream regulation mechanism of UBE2T, the miRDB database (http://www.mirdb.org/) was employed to predict the regulative miRNA of UBE2T. Subsequently, a total of 5 regulative miRNAs were predicted in the miRDB database (Table 1). miR-1305 and let-7c-3p exhibited the highest scores in the predicted results; thereby, it was speculated that miR-1305 might affect the development of LCSCs by targeting UBE2T. In addition, the biological prediction site miRDB (http://www.mirdb.org/) revealed that miR-1305 could target the UBE2T gene (Figure 4A).

**Table 1:** UBE2T Regulatory miRNA Prediction

Target Detail	Target Rank	Target Score	miRNA	Gene Symbol	Gene Description
Details	1	76	hsa-let-7c-3p	UBE2T	ubiquitin-conjugating enzyme E2T (putative)
Details	2	76	hsa-miR-1305	UBE2T	ubiquitin-conjugating enzyme E2T (putative)
Details	3	65	hsa-miR-3671	UBE2T	ubiquitin-conjugating enzyme E2T (putative)
Details	4	65	hsa-miR-5580-3p	UBE2T	ubiquitin-conjugating enzyme E2T (putative)
Details	5	62	hsa-miR-212-5p	UBE2T	ubiquitin-conjugating enzyme E2T (putative)

UBE2T, ubiquitin-conjugating enzyme E2T; miRNA, microRNA.

Next, a dual luciferase reporter gene assay was used to verify the aforementioned binding relationship. The results showed that there were no significant changes in luciferase activity in the cells transfected with UBE2T-mutation (mut) in the presence of miR-1305 mimic (p > 0.05). However, the luciferase activity in cells co-transfected with UBE2T-wild-type (WT) and miR-1305 mimic was found to significantly decrease (p < 0.05) (Figure 4B). Additionally, in order to understand the regulatory effect of miR-1305 on the UBE2T gene, the expression of UBE2T in LCSCs was detected by means of qRT-PCR and western blot analysis. The results showed that the expression of UBE2T in LCSCs transfected with miR-1305 mimic was significantly lower than that in LCSCs transfected with miR-1305 scramble (p < 0.05) (Figures 4C–4E). The results suggested that UBE2T was a target gene of miR-1305 and overexpression of miR-1305 could inhibit the expression of UBE2T.

### Restoration of miR-1305 Reversed the Promotion of LCSC Self-Renewal and Tumorigenicity Induced by UBE2T

**Figure 5:**
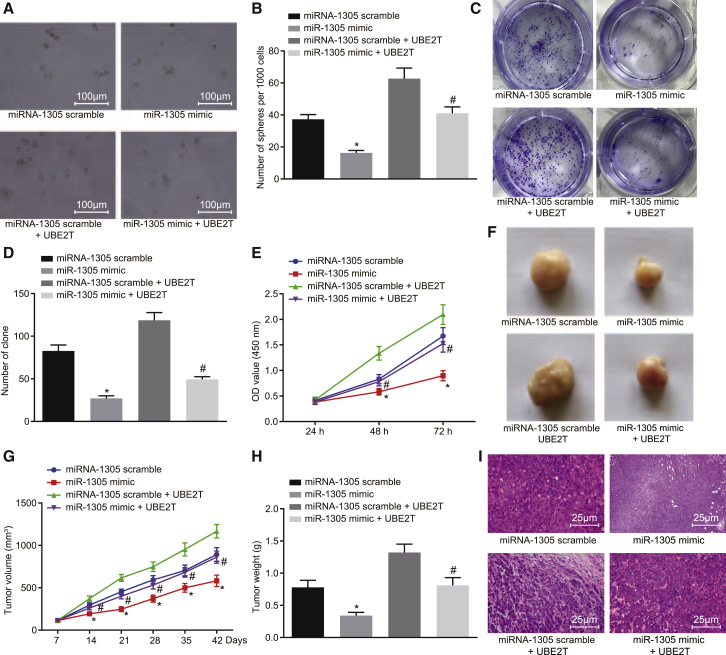
Upregulated miR-1305 Reduced LCSC Self-Renewal, Tumorigenesis, and Tumor Growth by Downregulating UBE2T (A) The sphere formation of LCSCs transfected with miR-1305 mimic with or without UBE2T (100×). (B) The quantitative analysis of (A). (C) The colony formation of LCSCs transfected with miR-1305 mimic with or without UBE2T. (D) The quantitative analysis of colony formation ability in (C). (E) The proliferative ability of LCSCs transfected with miR-1305 mimic with or without UBE2T, assessed by CCK-8 assay. (F) The observation of tumors in nude mice. (G) The tumor volume in nude mice injected with miR-1305 mimic-transfected or miR-1305 mimic + UBE2T-co-transfected LCSCs measured by Vernier caliper. (H) The tumor weight in nude mice injected with miR-1305 mimic-transfected or miR-1305 mimic + UBE2T-co-transfected LCSCs. (I) The pathological characteristics of tumor tissues in nude mice observed by H&E staining (400×). miR-1305 scramble group, LCSCs transfected with miR-1305 scramble or nude mice inoculated with miR-1305 scramble-transfected LCSCs; miR-1305 mimic group, LCSCs transfected with miR-1305 mimic or nude mice treated with miR-1305 mimic-transfected LCSCs; miR-1305 scramble + UBE2T group, LCSCs co-transfected with miR-1305 scramble and UBE2T or nude mice inoculated with co-transfected LCSCs; miR-1305 scramble + UBE2T group, LCSCs co-transfected with miR-1305 mimic and miR-1305 scramble or nude mice inoculated with co-transfected LCSCs. *p < 0.05 versus LCSCs transfected with miR-1305 scramble or nude mice inoculated with or miR-1305 scramble-transfected LCSCs; #p < 0.05 versus LCSCs co-transfected with miR-1305 scramble and UBE2T or nude mice inoculated with co-transfected LCSCs. Statistical data were described as mean ± SD. Data were analyzed by one-way ANOVA, and data in (E) and (G) were compared by repeated-measures ANOVA. The experiment was repeated 3 times independently (n = 6).

To further elucidate the mechanism underlying the negative regulation of LCSC self-renewal by miR-1305, we investigated the sphere formation, colony formation, and proliferation after restoring the UBE2T expression in the LCSCs overexpressing miR-1305. The LCSCs were co-transfected with miR-1305 mimic and UBE2T plasmid or miR-1305 scramble. The sphere formation and colony formation abilities of LCSCs transfected with miR-1305 mimic were found to significantly decrease, with weakened refractive index and irregular shape, compared to those transfected with miR-1305 scramble (p < 0.05). LCSCs co-transfected with miR-1305 mimic and UBE2T presented with diminished sphere formation and colony formation abilities of LCSCs, with weakened refractive index and irregular shapes compared to those co-transfected with miR-1305 scramble and UBE2T (Figures 5A–5D). In addition, the results of the CCK-8 assay revealed that the proliferative ability of LCSCs was markedly reduced by miR-1305 overexpression (p < 0.05). Moreover, the proliferative ability of LCSCs after UBE2T restoration in the presence of miR-1305 mimic was found to significantly decrease compared to those treated with miR-1305 scramble and UBE2T (p < 0.05) (Figure 5E).

We further compared the subcutaneous tumorigenesis of LCSC miR-1305 mimic versus LCSC miR-1305 scramble-derived grafts *in vivo* (Figures 5F–5H). We found that tumor volumes and weights significantly decreased by miR-1305 restoration. Furthermore, upregulation of UBE2T in the presence of miR-1305 mimic repressed tumor volumes and weights compared to miR-1305 scramble treatment with UBE2T upregulation. The results of H&E staining showed that the tumor tissue was in a lump and the nucleus was large and deeply stained; the tumor cells grew vigorously with more mitotic appearance and focal necrosis, the tumor tissue infiltrated the surrounding tissue, and it could invade muscle tissue in miR-1305 scramble-treated LCSCs. Meanwhile, miR-1305 mimic-treated LCSCs showed lighter nuclear staining and reduced nuclear division of tumor cells, with extensive necrosis in the tumor tissues and proliferated fibrous tissues around the necrotic area in different degrees. With UBE2T treatment in the presence of miR-1305 scramble, the tumor cells grew more vigorously, and mitosis became more obvious with no obvious necrotic areas in tumor tissue and infiltrated muscle tissue. Following miR-1305 mimic treatment with UBE2T, the nuclei staining of tumor cells became lighter, mitosis was attenuated, tumor tissue was necrotic, and fibrous tissue around the necrotic area was proliferated to different degrees (Figure 5I). Taken together, all the findings above demonstrated that restoration of miR-1305 inhibited HCC tumor growth *in vitro* and *in vivo*, primarily due to the suppression of UBE2T.

### miR-1305 Regulated Self-Renewal and Tumorigenic Potential of LCSCs through UBE2T-Mediated Akt-Signaling Pathway

**Figure 6:**
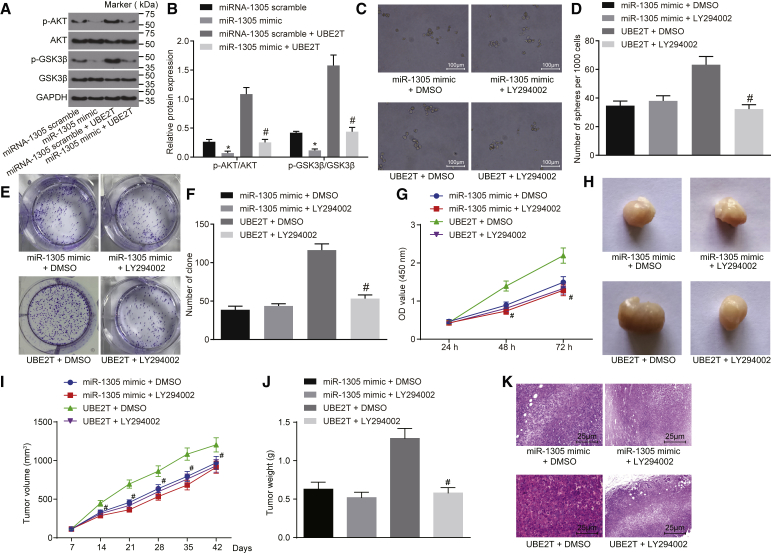
Self-Renewal and Tumorigenic Potential of LCSCs Were Repressed by miR-1305 through the UBE2T-Mediated Akt-Signaling Pathway (A) The gray value of Akt and GSK3β and their phosphorylation protein bands in LCSCs measured by western blot analysis. (B) The protein expressions of Akt and GSK3β and the extents of their phosphorylation in LCSCs, measured by western blot analysis. (C) The sphere formation assay in LCSCs treated with DMSO or LY294002 in the presence of miR-1305 mimic or UBE2T over-expression (100×). (D) The quantitative analysis of sphere formation ability in (C). (E) The soft agar colony formation assay in LCSCs treated with DMSO or LY294002 in the presence of miR-1305 mimic or UBE2T over-expression. (F) The quantitative analysis of soft agar colony formation in (E). (G) The proliferative ability of LCSCs that overexpressed miR-1305 or UBE2T and were treated with DMSO or LY294002, evaluated by CCK-8 assay. (H) Naked eye observation of tumor in nude mice treated with DMSO or LY294002 in the presence of miR-1305 mimic or UBE2T overexpression. (I) The tumor volume curve in nude mice treated with DMSO or LY294002 in the presence of miR-1305 mimic or UBE2T overexpression, measured by a Vernier caliper. (J) The tumor weight in nude mice in the presence of miR-1305 mimic or UBE2T overexpression with DMSO or LY294002 treatment. (K) The pathological characteristics of tumor in nude mice injected with miR-1305 mimic or UBE2T overexpression with DMSO or LY294002 treatment, observed with H&E staining (400×). miR-1305 scramble + DMSO group, LCSCs or nude mice transfected with miR-1305 mimic, then treated with DMSO; miR-1305 mimic + LY294002 group, LCSCs or nude mice transfected with miR-1305 mimic, then treated with LY294002; UBE2T + DMSO group, LCSCs or nude mice transfected with UBE2T overexpression, then treated with DMSO; UBE2T + LY294002 group, LCSCs or nude mice transfected with UBE2T overexpression, then treated with LY294002. *p < 0.05 versus LCSCs or nude mice transfected with miR-1305 mimic, then treated with DMSO; #p < 0.05 versus LCSCs or nude mice transfected with UBE2T overexpression, then treated with DMSO. Statistical data were described as mean ± SD. Data were analyzed by one-way ANOVA, and data in (G) and (I) were compared by repeated-measures ANOVA. The experiment was repeated 3 times independently (n = 6).

As aforementioned, we found that miR-1305 inhibited the self-renewal and tumorigenic potential of LCSCs by inhibiting the expression of UBE2T. Therefore, we aimed to further explore whether restoration of miR-1305 inhibited HCC tumor growth and attenuated the Akt-signaling pathway and whether decreased Akt signaling was mediated by UBE2T. The expression of Akt-signaling pathway-related genes was determined in the LCSCs overexpressing miR-1305 alone or both miR-1305 and UBE2T. The results showed the Akt-signaling pathway was indeed suppressed by miR-1305 in LCSCs, as demonstrated by reduced levels of phospho-Akt (p-Akt)/Akt and p-GSK3β/GSK3β. The levels of p-Akt/Akt and p-GSK3β/GSK3β in LCSCs were found to significantly diminish by co-transfection of miR-1305 and UBE2T relative to co-transfection with miR-1305 scramble and UBE2T (p < 0.05) (Figures 6A and 6B). Therefore, the data suggested that miR-1305 impeded the Akt-signaling pathway by targeting UBE2T.

In addition, an Akt inhibitor, LY294002, was employed to block the Akt-signaling pathway, with DMSO serving as a control. The sphere and colony formation abilities of LCSCs treated with miR-1305 mimic and LY294002 exhibited no significant differences compared to LCSCs treated with miR-1305 mimic and DMSO (p > 0.05). The sphere and colony formations of LCSCs treated with LY294002 in the presence of UBE2T were noted to significantly decrease, with an irregular shape and weak refractive index compared to those treated with DMSO in the presence of UBE2T (p < 0.05). Similarly, the results of the CCK-8 assay showed that the proliferative ability of LCSCs treated with miR-1305 mimic and LY294002 was not significantly different compared to LCSCs treated with miR-1305 mimic and DMSO (p > 0.05), but that of UBE2T-treated LCSCs was found to significantly decrease by LY294002 treatment (p < 0.05) (Figures 6C–6G).

The nude mice injected with the cells that overexpressed miR-1305 or UBE2T were treated with LY294002 or DMSO, so as to explore the role of the Akt-signaling pathway in the regulation of miR-1305 and UBE2T in tumor formation and growth. As expected, there were no significant changes in tumor volume and weight in mice injected with miR-1305 mimic-transfected cells when treated with LY294002 compared to those treated with DMSO (all p > 0.05); but, the tumor volume and weight significantly decreased by LY294002 treatment in the mice injected with UBE2T-transfected cells (all p < 0.05) (Figures 6H–6J). The degree of necrosis and infiltration of tumor in the mice injected with cells that overexpressed miR-1305 and LY294002 was not significantly different compared to those injected with cells that overexpressed miR-1305 and DMSO (p > 0.05). The treatment of LY294002 resulted in lighter nuclear staining and reduced nuclear division of tumor cells, with extensive necrosis in the tumor tissues and proliferated fibrous tissues around the necrotic area to different degrees in the mice injected with UBE2T-transfected cells (p < 0.05) (Figure 6K). These findings revealed that the miR-1305/UBE2T/Akt axis was involved in regulating LCSC self-renewal and tumorigenic potential as well as tumor growth *in vivo*.

## Discussion

Liver cancer is the fifth most commonly diagnosed tumor and the second most frequent cause of cancer-related deaths in men. Among primary liver cancers, HCC represents the major histological subtype, accounting for 70%–85% of cases of primary liver cancer.^20^ Similar to normal stem cells, CSCs are assumed to possess the abilities of self-renewal and production of differentiated cells.^21^ Even though CSC theory has gained much attention over the last few years, and suppression of CSCs is generally believed to be highly effective in impeding tumor progression, the regulatory mechanisms of CSCs remain unclear.^22^ The current study identified that miR-1305 was a novel miRNA that could specifically suppress the LCSCs’ stemness and HCC tumorigenesis by directly inhibiting UBE2T. Furthermore, we also demonstrated that the restoration of miR-1305 inhibited overall HCC tumorigenesis through the Akt-signaling pathway by binding to UBE2T. The result indicated that miR-1305 plays an important role in human HCC pathology and treatment and could potentially open new and viable modes of treatment in the future.

In comparison with previous studies on miRNAs and genes in LCSCs,^23^, ^24^ the current study illustrated different mechanisms. For instance, Liu et al.^23^ found that MHCC-97H cells in the absence of miR-155 exhibit a decreased number of CD90^+^CD133^+^ cells, decreased Oct4 expression, and a weakened sphere formation ability. Meanwhile, overexpression of miR-150 profoundly repressed cell proliferation and led to partial dispersion of spheres in CD133^+^ HCC cells.^24^ Herein, we observed a new mechanism by which miR-1305 exerts its inhibitory function on the stemness of LCSCs, by directly suppressing the expression of UBE2T at a post-transcriptional level. In addition, a previous study showed that UBE2T was a target gene of miR-543 and also could accelerate HCC growth by the mediation of p53, but data for the interaction between the p53 and UBE2T proteins were not shown in this study.^25^ The focus of the above study was changes of apoptosis-related factor p53 in HCC, while we focused on the changes of activation-related signal Akt in HCC cells. However, both studies revealed that UBE2T was as an oncogene in HCC. Furthermore, the current study not only added miR-1305 to the current list of specific miRNAs that suppress the stemness of LCSCs but also indicated toward the existence of a direct correlation between miR-1305 and UBE2T. By suppression of UBE2T and its downstream Akt-signaling pathway, miR-1305 impaired LCSCs’ stemness and overall HCC tumor growth.

One central finding in the current study was that UBE2T presented with a higher expression in HCC cell lines and LCSCs (CD133^+^CD13^+^ cells) than in the normal hepatic epithelial cell line. A previous study emphasized the important roles of the ubiquitin-proteasome pathway, which is a complex protein degradation system and plays in a wide range of biological processes, such as cell cycle control, signaling transduction, and tumorigenesis.^26^ Recently, enhanced expression of UBE2T has been noted in several tumors, such as breast and prostate cancers, and it serves as an attractive therapeutic target.^9^, ^10^ In addition, the results provided by Hu et al.^17^ verified that UBE2T elevated the colony formation and proliferation of nasopharyngeal carcinoma cells both *in vitro* and *in vivo*, which is in line with our findings that the silencing of UBE2T could diminish the LCSCs’ sphere and colony formation and proliferation abilities.

The current study also demonstrated that miR-130 acts as a tumor suppressor in HCC by attenuating the Akt-signaling pathway. The Akt-signaling pathway is well documented and known to be involved in cell proliferation and apoptosis, and thus it affects the progression of different kinds of tumors.^27^ A recent report found that HCC patients presenting with high levels of CXCR2 and CXCL5 showed an activated state of the Akt/GSK3β-signaling pathway.^28^ More specifically, an activated Akt/GSK3β-signaling pathway was associated with CSC migration.^29^ Similarly, it has been proven that the blockage of the Akt-signaling pathway significantly reduced the expression of Nanog, the neoplastic engine driving oncogenesis, and restored the activity of GSK3β.^30^, ^31^ Moreover, Akt suppression could diminish the self-renewal and the growth of CD133^+^ glioma CSCs.^16^ In the current study, the protein expressions of Akt and GSK3β and the extents of their phosphorylation were found to be decreased in cells treated with miR-1305 overexpression, while opposite trends were noted in cells treated with overexpressed UBE2T. The result suggested that the upregulation of miR-1305 could inhibit the activation of the Akt-signaling pathway, which was reversed by UBE2T.

**Figure 7:**
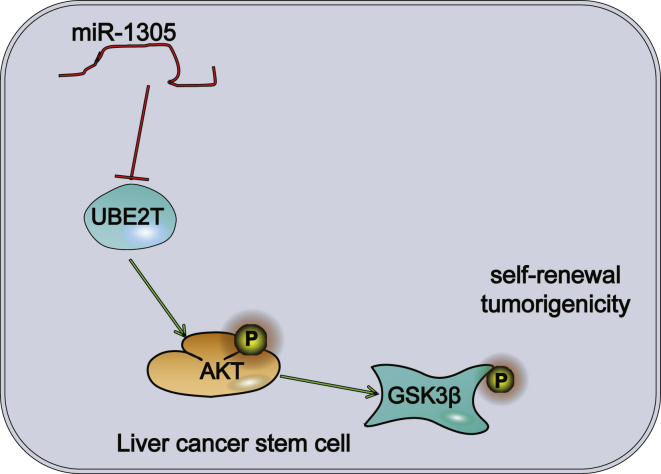
The Regulatory Mechanism of miR-1305 in the Self-Renewal and Proliferation of LCSCs miR-1305 can downregulate the Akt-signaling pathway mediated by UBE2T, thus inhibiting the proliferation, colony formation, sphere formation, and tumorigenic potential of LCSCs.

In summary, we demonstrated that UBE2T was overexpressed while miR-1305 was poorly expressed in HCC. In addition, we identified miR-1305 as a key regulator of proliferation, self-renewal, sphere and colony formations, as well as tumorigenicity via activation of the Akt-signaling pathway in LCSCs (Figure 7). Our data strongly suggested that miR-1305 could be used as a prognostic marker for HCC and serves as a potential therapeutic target. However, additional descriptions of the downregulation mechanism of miR-1305 in HCC are warranted, especially the molecules that bind to LCSCs when regulating their behavior. Besides, there must be other target genes for miR-1305, but our study shows a close relationship between UBE2T and miR-1305 under a series of constraints.

## Materials and Methods

### Ethics Statement

The investigation was conducted in accordance with the ethical standards of national and international guidelines, and it has been approved by the institutional review board of Jiangxi Cancer Hospital. Great efforts were made to minimize the number and suffering of the included animals.

### Microarray-Based Gene Expression Analysis

The GEO database (https://www.ncbi.nlm.nih.gov/geo/) was used to obtain the microarray of HCC. Next, the R language limma package was used to analyze the DEGs, with |log fold change (FC)| > 2 and p < 0.05 serving as the screening criteria. A heatmap of DEGs was plotted using the pheatmap package. Then, HCC-related genes were retrieved from the MalaCards database (https://www.malacards.org/), and the gene interaction network was plotted in the STRING database (https://string-db.org/). Then, the expression of UBE2T in the HCC samples collected in TCGA was analyzed based on the UALCAN database (http://ualcan.path.uab.edu/cgi-bin/ualcan-res.pl). Finally, miRDB (http://www.mirdb.org/), miRTarBase (http://mirtarbase.mbc.nctu.edu.tw/php/search.php), and TargetScan (http://www.targetscan.org/vert_71/) were used to predict the miRNAs that regulated UBE2T.

### Cell Lines and Cell Culture

HCC cell lines, HCCLM3 and HepG2, were purchased from Shanghai Institutes for Biological Sciences, Chinese Academy of Sciences (Shanghai, China). A normal liver epithelial cell line, LO2, and other two HCC cell lines, Hep3B and Huh7, were purchased from American Type Culture Collection (Manassas, VA, USA). All four HCC cell lines were cultured in DMEM (Invitrogen, Carlsbad, CA, USA) containing 10% fetal bovine serum (FBS; 10099141; Gibco, Carlsbad, CA, USA). The normal liver epithelial cell line LO2 was cultured in bronchial epithelial growth medium (ClonetIcs, Walkersville, MD, USA) containing epithelial growth factor (5 ng/mL) and phosphoethanolamine (70 ng/mL). Finally, the cells were cultured in a humidified incubator at 37°C with 5% CO_2_ in air (thromo3111, Jinan Bisheng Medical Devices, Jinan, Shandong, China). The culture medium was replaced at the second day, and the cells were sub-cultured at the third to fourth day.^32^

### LCSC Sorting and Identification

The HCC cell line Huh7 was incubated with DMEM supplemented with 10% FBS in a humidified incubator at 37°C with 5% CO_2_ in air. When cell confluence reached 70%–80%, the cells were detached with 0.25% trypsin and were prepared into a single-cell suspension. The cell suspension was transferred into a 1.5-mL Eppendorf (EP) tube. Then, 5 μL antibodies of CD133 (ab19898, dilution ratio of 1:300) and CD13 (ab227663, dilution ratio of 1:100) were added for incubation at 4°C for 30 min, avoiding exposure to light. Both antibodies were purchased from Abcam (Cambridge, MA, USA). Following centrifugation at 700 rpm for 5 min, the supernatant was discarded. Flow cytometry was carried out using an Accuri C6 flow cytometer (Becton Dickinson, Franklin Lakes, NJ, USA), and then CD13^+^CD133^+^ cells were isolated and identified as LCSCs.^1^, ^33^

### Cell Transfection and Treatment

The small interfering RNAs (siRNAs) targeting UBE2T (silencer [si]-UBE2T), miR-1305 mimic, UBE2T overexpression plasmid (UBE2T), and their controls (si-NC and miR-1305 scramble) were purchased from Guangzhou RiboBio (Guangzhou, Guangdong, China). LY294002 (10 μM, Sigma-Aldrich, St. Louis, MO, USA), an Akt inhibitor, was employed to block the Akt-signaling pathway, with DMSO (10 μM, Sigma-Aldrich, St. Louis, MO, USA) as a control.^34^ The steps of cell transfection were as follows: an appropriate amount of cells was maintained in a 24-well plate; when cell density reached 50%–60%, the LCSCs (CD13^+^CD133^+^ and CD13^−^CD133^−^ cells) were transfected instantly with liposome Lipofectamine 2000 (Invitrogen, Carlsbad, CA, USA). Subsequent experiments were conducted after transfection for 24 h.

### RNA Isolation and Quantification

Total RNA content from the cells was extracted using a Trizol reagent (16096020, Thermo Fisher Scientific, Waltham, MA, USA). Then, the total RNA was reversely transcribed into cDNA using a cDNA synthesis kit (K1622; Fermentas, Burlington, ON, Canada). The expression of miR-1305 was determined by TaqMan miRNA assay (Ambion, Austin, TX, USA), with U6 as an endogenous control. The expressions of UBE2T, CD133, CD13, Nanog, Sox2, and Oct-4 were determined by PrimeScript RT-PCR kits (TaKaRa, Shiga, Japan), with glyceraldehyde-3-phosphate dehydrogenase (GAPDH) serving as an endogenous control. The primers used are listed in Table 2, and the fold changes were calculated by means of relative quantification (2^−ΔΔCt^ method).

**Table 2:** Primer Sequences for qRT-PCR

Gene	Forward (5′–3′)	Reverse (5′–3′)
miR-1305	ACAGGCCGGGACAAGTGCAATA	GCTGTCAACGATACGCTACGTAACG
U6	AACGCTTCACGAATTTGCGT	CTCGCTTCGGCAGCACA
UBE2T	ATCCCTCAACATCGCAACTGT	CAGCCTCTGGTAGATTATCAAGC
Nanog	CAGAAGGCCTCAGCACCTAC	ATTGTTCCAGGTCTGGTTGC
Sox2	AGCTACAGCATGATGCAGGA	GGTCATGGAGTTGTACTGCA
Oct4	CAGTGCCCGAAACCCACAC	GGAGACCCAGCAGCCTCAAA
GAPDH	ATGGAGAAGGCTGGGGCTC	AAGTTGTCATGGATGACCTTG

miR-1305, microRNA-1305; UBE2T, ubiquitin-conjugating enzyme E2T; GAPDH, glyceraldehyde-3-phosphate dehydrogenase.

### Western Blot Analysis

The total protein content from the cells was extracted using a radio-immunoprecipitation assay (RIPA) lysis buffer (R0010; Beijing Solabio Life Sciences, Beijing, China) containing PMSF. The total protein concentration was determined by bicinchoninic acid (BCA) kit. A total of 50 μg protein was dissolved in 2× SDS buffer loading and was separated by 10% SDS- PAGE, and then it was transferred onto a polyvinylidene fluoride (PVDF) membrane by the wet-transfer method. The membranes were blocked with 5% skim milk for 1 h at room temperature, and then they were incubated with the following diluted primary antibodies: mouse monoclonal antibody against GAPDH (ab8245, dilution ratio of 1:10,000), mouse monoclonal antibody against UBE2T (H00029089-M02, dilution ratio of 1:500), rabbit polyclonal antibody against Sox2 (ab97959, dilution ratio of 1:10,000), rabbit polyclonal antibody against Oct4 (ab18976, dilution ratio of 1:1,000), Nanog (ab21624, dilution ratio of 1:200), rabbit polyclonal antibody against Akt (ab179463, dilution ratio of 1:10,000), rabbit polyclonal antibody against p-Akt-S129 (ab133458, dilution ratio of 1:500), mouse monoclonal antibody against GSK3β (ab93926, dilution ratio of 1:1,000), and rabbit polyclonal antibody against p-GSK3β (phospho Y216) (ab75745, dilution ratio of 1:1,000) at 4°C overnight. All the aforementioned antibodies were purchased from Abcam (Cambridge, MA, USA), except UBE2T (Santa Cruz Biotechnology, Santa Cruz, CA, USA).

After rinsing 3 times with Tris-buffered saline with Tween-20 (TBST), the membranes were then incubated with horseradish peroxidase (HRP)-conjugated secondary antibody for 1 h. The solution A and solution B from the enhanced chemiluminescence (ECL) fluorescent detection kit (BB-3501, Amersham Pharmacia, Piscataway, NJ, USA) were dripped onto the membranes and exposed to the gel imager. The images were visualized by a Bio-Rad image analysis system (Bio-Rad, Hercules, CA, USA). The relative protein expression was expressed by the gray value of the target protein band to that of the GAPDH protein band using the Quantity One version (v.)4.6.2 software.

### Sphere Formation Assay

Adherent LCSCs were trypsinized, and a single-cell suspension was subsequently prepared. The cells (1 × 10^3^ cells/mL) were inoculated into ultra-low attachment 24-well plates and cultured with serum-free medium (2 mL/well). Suspended spheres were obtained and half of the medium was refreshed every other day. After 10 days of continuous culture, the newly formed spheres were microscopically observed and photographed. Subsequently, the number of spheres newly formed in each well was counted and recorded.

### Soft Agar Colony Formation Assay

After autoclaving, 0.7% agar with low melting point was freshly prepared with DMEM and stored at 4°C for further use. Subsequently, 0.7% agarose was heated and melted, and 2 mL agar was spread evenly in the 100-mm dishes and solidified for further use.

The cell suspension (1 mL) and 0.7% agarose solution (1 mL) were mixed into 0.35% agarose cell mixture. The cell mixture was inoculated into a Petri dish in triplicate at a density of 1 × 10^4^ cells/100 cm^2^. After the upper layer of agar solidified, the surface of the agar was gently added with 2–3 mL culture medium to avoid crushing. The cells were then incubated at 37°C with 5% CO_2_ in air. The medium was renewed every 2 days, and then the culture was terminated after 1 month. The colonies formed in the Petri dish were observed under an inverted microscope, and the average value was obtained. A mass consisting of more than 50 cells was regarded as one colony.

### CCK-8 Assay

A total of 100 μL cells was seeded in a 96-well plate at a density of about 2,000 cells/well. The cells that were incubated at 37°C were added with 10 μL CCK-8 solution (35000, AAT Bioquest, Sunnyvale, CA, USA) in each well at 24, 48, and 72 h. After another 4-h incubation at 37°C, the optical density (OD) value of each well was measured using a microplate reader (Thermo Fisher Scientific, Waltham, MA, USA) at the wavelength of 450 nm, and the proliferative ability of LCSCs was calculated and recorded.

### Tumor Xenograft in Nude Mice

A total of 60 BALB/c nude mice of specific-pathogen-free (SPF) grade (aged 3–4 weeks; 30 males and 30 females; purchased from Shanghai SLAC Laboratory Animal, Shanghai, China) was maintained at a temperature of 25°C–27°C and a relative humidity of 45%–50%. Briefly, approximately 1 × 10^6^ cells (25 μL cell suspension) were injected subcutaneously under the liver capsule of the nude mice. On days 7, 14, 21, 28, 36, and 42 after inoculation,^32^ the length (L) and width (W) of the tumors were measured using a digital caliper, and the volume (V) was calculated as follows: V = L × W^2^ × 0.5;^35^ the growth curve was plotted. After 6 weeks of inoculation, all nude mice were euthanized and the tumors were excised and weighed. Tumor tissues were fixed with 4% formaldehyde, paraffin embedded, and stained with H&E for pathological analysis. The operation was carried out according to the instructions of the H&E kit (Beyotime Biotechnology, Shanghai, China).^36^

### Dual Luciferase Reporter Gene Assay

A dual luciferase reporter gene assay was employed in order to verify whether UBE2T was a direct target of miR-1305. In detail, a synthetic UBE2T 3′ UTR gene fragment was introduced into a pMIR-reporter (Beijing Huayueyang Biotechnology, Beijing, China) using endonuclease sites Spe I and Hind III. The complementary mut site of the seed sequence was designed on the UBE2T WT sequence. The target fragment was inserted into the pMIR-reporter plasmid by restriction endonuclease digestion and T4 DNA ligase. The correctly sequenced recombinant luciferase reporter plasmids UBE2T-WT and UBE2T-mut were co-transfected with miR-1305 mimic into HEK293T cells (CRL-1415, Shanghai Xin Yu Biotech, Shanghai, China). After 48 h of transfection, the cells were collected and lysed. Using a luciferase assay kit (RG005, Shanghai Beyotime Biotechnology, Shanghai, China), the luciferase activity was measured on a Glomax20/20 luminometer (Promega, Madison, WI, USA).

### Statistical Analysis

Statistical analyses were processed using the SPSS 21.0 statistical software (IBM, Armonk, NY, USA). Measurement data were expressed as mean ± SD. Initially, normal distribution and homogeneity of variance were tested for all data. For the data with normal distribution and equal variances, a paired t test was employed for intra-group comparisons, and a non-paired t test was applied to the statistical differences between two groups. Multi-group comparisons were conducted using the one-way ANOVA or the repeated-measures ANOVA, followed by Tukey’s post hoc test. The data with skew distribution or unequal variances were compared using the rank-sum test. Results were considered to be statistically significant at p < 0.05.

## Author Contributions

X.W. participated in the study design and experimental work. X.Y. and J.Z. participated in sample collection and data analysis. X.W. and C.Z participated in the analysis and interpretation of the data and contributed to revising the manuscript. All authors approved the final manuscript.

## Conflicts of Interest

The authors declare no competing interests.

## Supplemental Information

Document S1. UBE2T Gene Descriptions

Document S2. Article plus Supplemental Information

## References

[1] Tang, H., Jin, Y., Jin, S., Tan, Z., Peng, Z., and Kuang, Y. (2016). Arsenite inhibits the function of CD133+ CD13+ liver cancer stem cells by reducing PML and Oct4 protein expression. Tumour Biol. 37, 14103-14115. 10.1007/s13277-016-5195-7 27517564

[2] Chen, W., Zheng, R., Baade, P.D., Zhang, S., Zeng, H., Bray, F., Jemal, A., Yu, X.Q., and He, J. (2016). Cancer statistics in China, 2015. CA Cancer J. Clin. 66, 115-132. 10.3322/caac.21338 26808342

[3] Ferlay, J., Soerjomataram, I., Dikshit, R., Eser, S., Mathers, C., Rebelo, M., Parkin, D.M., Forman, D., and Bray, F. (2015). Cancer incidence and mortality worldwide: sources, methods and major patterns in GLOBOCAN 2012. Int. J. Cancer 136, E359-E386. 10.1002/ijc.29210 25220842

[4] Fong, Z.V., and Tanabe, K.K. (2014). The clinical management of hepatocellular carcinoma in the United States, Europe, and Asia: a comprehensive and evidence-based comparison and review. Cancer 120, 2824-2838. 10.1002/cncr.28730 24897995

[5] Chen, Y., Yu, D., Zhang, H., He, H., Zhang, C., Zhao, W., and Shao, R.G. (2012). CD133(+)EpCAM(+) phenotype possesses more characteristics of tumor initiating cells in hepatocellular carcinoma Huh7 cells. Int. J. Biol. Sci. 8, 992-1004. 10.7150/ijbs.4454 PMC3421230 22904667

[6] Haraguchi, N., Ishii, H., Mimori, K., Tanaka, F., Ohkuma, M., Kim, H.M., Akita, H., Takiuchi, D., Hatano, H., Nagano, H., et al. (2010). CD13 is a therapeutic target in human liver cancer stem cells. J. Clin. Invest. 120, 3326-3339. 10.1172/JCI42550 PMC2929722 20697159

[7] Kohga, K., Tatsumi, T., Takehara, T., Tsunematsu, H., Shimizu, S., Yamamoto, M., Sasakawa, A., Miyagi, T., and Hayashi, N. (2010). Expression of CD133 confers malignant potential by regulating metalloproteinases in human hepatocellular carcinoma. J. Hepatol. 52, 872-879. 10.1016/j.jhep.2009.12.030 20395004

[8] Wang, Y., Leng, H., Chen, H., Wang, L., Jiang, N., Huo, X., and Yu, B. (2016). Knockdown of UBE2T Inhibits Osteosarcoma Cell Proliferation, Migration, and Invasion by Suppressing the PI3K/Akt Signaling Pathway. Oncol. Res. 24, 361-369. 10.3727/096504016X14685034103310 PMC7838603 27712593

[9] Ueki, T., Park, J.H., Nishidate, T., Kijima, K., Hirata, K., Nakamura, Y., and Katagiri, T. (2009). Ubiquitination and downregulation of BRCA1 by ubiquitin-conjugating enzyme E2T overexpression in human breast cancer cells. Cancer Res. 69, 8752-8760. 10.1158/0008-5472.CAN-09-1809 19887602

[10] Wen, M., Kwon, Y., Wang, Y., Mao, J.H., and Wei, G. (2015). Elevated expression of UBE2T exhibits oncogenic properties in human prostate cancer. Oncotarget 6, 25226-25239. 10.18632/oncotarget.4712 PMC4694827 26308072

[11] Yu, H., Xiang, P., Pan, Q., Huang, Y., Xie, N., and Zhu, W. (2016). Ubiquitin-Conjugating Enzyme E2T is an Independent Prognostic Factor and Promotes Gastric Cancer Progression. Tumour Biol. 37, 11723-11732. 10.1007/s13277-016-5020-3 27020591

[12] Pu, F., Chen, F., and Shao, Z. (2016). MicroRNAs as biomarkers in the diagnosis and treatment of chondrosarcoma. Tumour Biol. 37, 15433-15436. 10.1007/s13277-016-5468-1 27730542

[13] Zhang, L., Sun, X., Chen, S., Yang, C., Shi, B., Zhou, L., and Zhao, J. (2017). Long noncoding RNA DANCR regulates miR-1305-Smad 4 axis to promote chondrogenic differentiation of human synovium-derived mesenchymal stem cells. Biosci. Rep. 37, BSR20170347. 10.1042/BSR20170347 PMC5520215 28674107

[14] Chen, Z., and Liu, H.L. (2017). Restoration of miR-1305 relieves the inhibitory effect of nicotine on periodontal ligament-derived stem cell proliferation, migration, and osteogenic differentiation. J. Oral Pathol. Med. 46, 313-320. 10.1111/jop.12492 27604968

[15] Jin, S., Collin, J., Zhu, L., Montaner, D., Armstrong, L., Neganova, I., and Lako, M. (2016). A Novel Role for miR-1305 in Regulation of Pluripotency-Differentiation Balance, Cell Cycle, and Apoptosis in Human Pluripotent Stem Cells. Stem Cells 34, 2306-2317. 10.1002/stem.2444 PMC5031214 27339422

[16] Wei, Y., Jiang, Y., Zou, F., Liu, Y., Wang, S., Xu, N., Xu, W., Cui, C., Xing, Y., Liu, Y., et al. (2013). Activation of PI3K/Akt pathway by CD133-p85 interaction promotes tumorigenic capacity of glioma stem cells. Proc. Natl. Acad. Sci. USA 110, 6829-6834. 10.1073/pnas.1217002110 PMC3637720 23569237

[17] Hu, W., Xiao, L., Cao, C., Hua, S., and Wu, D. (2016). UBE2T promotes nasopharyngeal carcinoma cell proliferation, invasion, and metastasis by activating the AKT/GSK3β/β-catenin pathway. Oncotarget 7, 15161-15172. 10.18632/oncotarget.7805 PMC4924777 26943030

[18] Perez-Peña, J., Corrales-Sanchez, V., Amir, E., Pandiella, A., and Ocana, A. (2017). Ubiquitin-conjugating enzyme E2T (UBE2T) and denticleless protein homolog (DTL) are linked to poor outcome in breast and lung cancers. Sci. Rep. 7, 17530. 10.1038/s41598-017-17836-7 PMC5727519 29235520

[19] Gong, Y.Q., Peng, D., Ning, X.H., Yang, X.Y., Li, X.S., Zhou, L.Q., and Guo, Y.L. (2016). UBE2T silencing suppresses proliferation and induces cell cycle arrest and apoptosis in bladder cancer cells. Oncol. Lett. 12, 4485-4492. 10.3892/ol.2016.5237 PMC5228076 28101210

[20] Yamashita, T., and Wang, X.W. (2013). Cancer stem cells in the development of liver cancer. J. Clin. Invest. 123, 1911-1918. 10.1172/JCI66024 PMC3635728 23635789

[21] Kim, H.M., Haraguchi, N., Ishii, H., Ohkuma, M., Okano, M., Mimori, K., Eguchi, H., Yamamoto, H., Nagano, H., Sekimoto, M., et al. (2012). Increased CD13 expression reduces reactive oxygen species, promoting survival of liver cancer stem cells via an epithelial-mesenchymal transition-like phenomenon. Ann. Surg. Oncol. 19 (Suppl 3), S539-S548. 10.1245/s10434-011-2040-5 21879266

[22] Chang, Y.L., Zhou, P.J., Wei, L., Li, W., Ji, Z., Fang, Y.X., and Gao, W.Q. (2015). MicroRNA-7 inhibits the stemness of prostate cancer stem-like cells and tumorigenesis by repressing KLF4/PI3K/Akt/p21 pathway. Oncotarget 6, 24017-24031. 10.18632/oncotarget.4447 PMC4695167 26172296

[23] Liu, F., Kong, X., Lv, L., and Gao, J. (2015). MiR-155 targets TP53INP1 to regulate liver cancer stem cell acquisition and self-renewal. FEBS Lett. 589, 500-506. 10.1016/j.febslet.2015.01.009 25601564

[24] Zhang, J., Luo, N., Luo, Y., Peng, Z., Zhang, T., and Li, S. (2012). microRNA-150 inhibits human CD133-positive liver cancer stem cells through negative regulation of the transcription factor c-Myb. Int. J. Oncol. 40, 747-756. 10.3892/ijo.2011.1242 22025269

[25] Liu, L.P., Yang, M., Peng, Q.Z., Li, M.Y., Zhang, Y.S., Guo, Y.H., Chen, Y., and Bao, S.Y. (2017). UBE2T promotes hepatocellular carcinoma cell growth via ubiquitination of p53. Biochem. Biophys. Res. Commun. 493, 20-27. 10.1016/j.bbrc.2017.09.091 28935368

[26] Hao, J., Xu, A., Xie, X., Hao, J., Tian, T., Gao, S., Xiao, X., and He, D. (2008). Elevated expression of UBE2T in lung cancer tumors and cell lines. Tumour Biol. 29, 195-203. 10.1159/000148187 18667844

[27] Liu, L., Dong, Z., Liang, J., Cao, C., Sun, J., Ding, Y., and Wu, D. (2014). As an independent prognostic factor, FAT10 promotes hepatitis B virus-related hepatocellular carcinoma progression via Akt/GSK3β pathway. Oncogene 33, 909-920. 10.1038/onc.2013.236 23812429

[28] Zhou, S.L., Zhou, Z.J., Hu, Z.Q., Li, X., Huang, X.W., Wang, Z., Fan, J., Dai, Z., and Zhou, J. (2015). CXCR2/CXCL5 axis contributes to epithelial-mesenchymal transition of HCC cells through activating PI3K/Akt/GSK-3β/Snail signaling. Cancer Lett. 358, 124-135. 10.1016/j.canlet.2014.11.044 25462858

[29] Su, Y.J., Lin, W.H., Chang, Y.W., Wei, K.C., Liang, C.L., Chen, S.C., and Lee, J.L. (2015). Polarized cell migration induces cancer type-specific CD133/integrin/Src/Akt/GSK3β/β-catenin signaling required for maintenance of cancer stem cell properties. Oncotarget 6, 38029-38045. 10.18632/oncotarget.5703 PMC4741982 26515729

[30] Liu, C.W., Li, C.H., Peng, Y.J., Cheng, Y.W., Chen, H.W., Liao, P.L., Kang, J.J., and Yeng, M.H. (2014). Snail regulates Nanog status during the epithelial-mesenchymal transition via the Smad1/Akt/GSK3β signaling pathway in non-small-cell lung cancer. Oncotarget 5, 3880-3894. 10.18632/oncotarget.2006 PMC4116528 25003810

[31] Jeter, C.R., Liu, B., Liu, X., Chen, X., Liu, C., Calhoun-Davis, T., Repass, J., Zaehres, H., Shen, J.J., and Tang, D.G. (2011). NANOG promotes cancer stem cell characteristics and prostate cancer resistance to androgen deprivation. Oncogene 30, 3833-3845. 10.1038/onc.2011.114 PMC3140601 21499299

[32] Shi, Y., Qin, N., Zhou, Q., Chen, Y., Huang, S., Chen, B., Shen, G., and Jia, H. (2017). Role of IQGAP3 in metastasis and epithelial-mesenchymal transition in human hepatocellular carcinoma. J. Transl. Med. 15, 176. 10.1186/s12967-017-1275-8 PMC5558666 28810875

[33] Castelli, G., Pelosi, E., and Testa, U. (2017). Liver Cancer: Molecular Characterization, Clonal Evolution and Cancer Stem Cells. Cancers (Basel) 9, E127. 10.3390/cancers9090127 PMC5615342 28930164

[34] Cheng, W., Chen, W., Wang, P., and Chu, J. (2018). Asiatic acid protects differentiated PC12 cells from Aβ25-35-induced apoptosis and tau hyperphosphorylation via regulating PI3K/Akt/GSK-3β signaling. Life Sci. 208, 96-101. 10.1016/j.lfs.2018.07.016 30017668

[35] Kun-Peng, Z., Xiao-Long, M., and Chun-Lin, Z. (2017). LncRNA FENDRR sensitizes doxorubicin-resistance of osteosarcoma cells through down-regulating ABCB1 and ABCC1. Oncotarget 8, 71881-71893. 10.18632/oncotarget.17985 PMC5641097 29069754

[36] Wang, Y., Zhang, Y., Yang, T., Zhao, W., Wang, N., Li, P., Zeng, X., and Zhang, W. (2017). Long non-coding RNA MALAT1 for promoting metastasis and proliferation by acting as a ceRNA of miR-144-3p in osteosarcoma cells. Oncotarget 8, 59417-59434. 10.18632/oncotarget.19727 PMC5601743 28938647

