# Supplementary material for: MicroRNA-1305 Inhibits the Stemness of LCSCs and Tumorigenesis by Repressing the UBE2T-Dependent Akt-Signaling Pathway
Source: Mol Ther Nucleic Acids. 2019 Apr 22;16:721–32. doi: 10.1016/j.omtn.2019.04.013 (PMC6535505; doi:10.1016/j.omtn.2019.04.013)
Supplement: Document S1. UBE2T Gene Descriptions [file mmc1.pdf]

OMTN, Volume *16*

Supplemental Information

MicroRNA-1305 Inhibits the Stemness of LCSCs and Tumorigenesis by Repressing the UBE2T-Dependent Akt-Signaling Pathway

Xiaoyong Wei, Xiaolong You, Jianlong Zhang, and Cuncai Zhou

UBE2T Gene Descriptions:

NCBI GeneID：29089

GenBank Accession：NM_014176

NCBI Protein Accession：NP_054895

>NM_014176.4 Homo sapiens ubiquitin conjugating enzyme E2 T (UBE2T), transcript variant 1, mRNA

GTTGGTCCGCGCGTTGCTGCGTTGTGAGGGGTGTCAGCTCAGTGCATCCCAGGCAGCTC
TTAGTGTGGAGCAGTGAACTGTGTGTGGTTCCTTCTACTTGGGGATCATGCAGAGAGCT
TCACGTCTGAAGAGAGAGCTGCACATGTTAGCCACAGAGCCACCCCCAGGCATCACAT
GTTGGCAAGATAAAGACCAAATGGATGACCTGCGAGCTCAAATATTAGGTGGAGCCAA
CACACCTTATGAGAAAGGTGTTTTTAAGCTAGAAGTTATCATTCCTGAGAGGTACCCAT
TTGAACCTCCTCAGATCCGATTTCTCACTCCAATTTATCATCCAAACATTGATTCTGCTG
GAAGGATTTGTCTGGATGTTCTCAAATTGCCACCAAAAGGTGCTTGGAGACCATCCCTC
AACATCGCAACTGTGTTGACCTCTATTCAGCTGCTCATGTCAGAACCCAACCCTGATGA
CCCGCTCATGGCTGACATATCCTCAGAATTTAAATATAATAAGCCAGCCTTCCTCAAGA
ATGCCAGACAGTGGACAGAGAAGCATGCAAGACAGAAACAAAAGGCTGATGAGGAAG
AGATGCTTGATAATCTACCAGAGGCTGGTGACTCCAGAGTACACAACTCAACACAGAA
AAGGAAGGCCAGTCAGCTAGTAGGCATAGAAAAGAAATTTCATCCTGATGTTTAGGGG
ACTTGTCCTGGTTCATCTTAGTTAATGTGTTCTTTGCCAAGGTGATCTAAGTTGCCTACC
TTGAATTTTTTTTTAAATATATTTGATGACATAATTTTTGTGTAGTTTATTTATCTTGTAC
ATATGTATTTTGAAATCTTTTAAACCTGAAAAATAAATAGTCATTTAATGTTGA

>NP_054895.1 ubiquitin-conjugating enzyme E2 T isoform 1 [Homo sapiens]

MQRASRLKRELHMLATEPPPGITCWQDKDQMDDLRAQILGGANTPYEKGVFKLEVIIPER
YPFEPPQIRFLTPIYHPNIDSAGRICLDVLKLPPKGAWRPSLNIATVLTSIQLLMSEPNPDDPL
MADISSEFKYNKPAFLKNARQWTEKHARQKQKADEEEMLDNLPEAGDSRVHNSTQKRKA
SQLVGIEKKFHPDV
